# Supplementary material for: Colorectal cancer with Synchronous liver-limited Metastases: the protocol of an Inception Cohort study (CoSMIC)
Source: BMJ Open. 2017 Jun 9;7(6):e015018. doi: 10.1136/bmjopen-2016-015018 (PMC5734400; doi:10.1136/bmjopen-2016-015018)
Supplement: Author's manuscript [file bmjopen-2016-015018.draft_revisions.pdf]

BMJ Open

Colorectal cancer with Synchronous liver-limited Metastases: The protocol of an Inception Cohort study (CoSMIC).

For peer review only

Journal:	*BMJ Open*
Manuscript ID	bmjopen-2016-015018
Article Type:	Protocol
Date Submitted by the Author:	03-Nov-2016
Complete List of Authors:	Siriwardena, Ajith; Manchester Royal Infirmary, Manchester HepatoPancreatobiliary Centre<br>Chan, Anthony; Manchester Royal Infirmary, Department of Surgery<br>Ignatowicz, Agnieszka; Warwick Medical School, The University of Warwick, Division of Health Sciences<br>Mason, James; University of Warwick Warwick Medical School
**Primary Subject Heading**:	Surgery
Secondary Subject Heading:	Oncology
Keywords:	Gastrointestinal tumours < GASTROENTEROLOGY, Hepatobiliary disease < GASTROENTEROLOGY, Colorectal surgery < SURGERY, Hepatobiliary surgery < SURGERY

SCHOLARONE™
Manuscripts

Colorectal cancer with Synchronous liver-limited Metastases: The protocol of an Inception Cohort study (CoSMIC).

Ajith K Siriwardena MD FRCS[1,2], Anthony KC Chan MPhil MRCS[1,3], Agnieszka M Ignatowicz BSc MA PhD[3] and James M Mason DPhil[3] on behalf of the *CoSMIC study collaborators.

[1]Regional Hepato-Pancreato-Biliary Unit, Manchester Royal Infirmary, Oxford Road, Manchester, M13 9WL, UK

[2]Faculty of Medicine, University of Manchester, UK

[3]Warwick Medical School, University of Warwick, Coventry, CV4 7AL, UK

Correspondence to: Professor Ajith Siriwardena MD FRCS

Regional Hepato-Pancreato-Biliary Surgery Unit

Manchester Royal Infirmary

Oxford Road, Manchester M13 9WL.

Phone: +44 161 276 1234

Email: ajith.siriwardena@cmft.nhs.uk

Word Count (excluding title page, abstract, references, figures and tables): 4,072.

For peer review only

For peer review only - http://bmjopen.bmj.com/site/about/guidelines.xhtml

ABSTRACT

INTRODUCTION

Colorectal cancer is the fourth most common cancer in the UK and an important cause of cancer-related death. In 20% of patients, there is metastasis to the liver or beyond at the time of diagnosis. The management of synchronous disease is complex. Conventional surgery removes the colorectal primary first, followed by chemotherapy, with resection of the liver metastases as a final step. Advances in the availability and safety of liver surgery, anaesthesia and critical care have made two alternative options feasible. The first is synchronous resection of the primary and liver metastases. The second is resection of the metastatic disease as the first step, termed the reverse or liver-first approach.

Currently, evidence is inadequate to inform the selection of care pathway for patients with colorectal cancer with synchronous liver-limited hepatic metastases. Specifically, optimal pathways are not defined and there is a dearth of prospectively recorded cohort-defining factors influencing treatment selection or outcome.

METHODS AND ANALYSIS

CoSMIC is an inception cohort study of patients with a new diagnosis of colorectal cancer with synchronous liver-limited metastases. The sequence of treatment received by each patient, and factors influencing treatment decisions, will be evaluated against European Society of Medical Oncology guidelines. Clinical data will be collected, and the effect of surgery on quality of life, morbidity, mortality and long-term outcome will be compared for different treatment sequences adjusted for prognostic factors. Disease-free survival or progression will be measured at 1, 2 and 5 years. A nested qualitative study will also ascertain patient experiences and clinician perspectives on delivery of care.

For peer review only - http://bmjopen.bmj.com/site/about/guidelines.xhtml

DISSEMINATION

CoSMIC is the first prospective study directly comparing outcomes between the different surgical sequences of patients with colorectal cancer and liver-limited metastases, providing important outcome data on commonly used treatment pathways including for the first time from a single inception point.

Strengths and limitations of this study

- First prospective study directly comparing outcomes between the different surgical sequences of patients with colorectal cancer and liver-limited metastases
- Completion of the CoSMIC study protocol will provide important new evidence about the treatment of patients with colorectal cancer with synchronous liver-limited metastases and provide objective evidence to guide future studies (including randomised evaluations) in this area.
- The variables involved in the treatment allocation of such patients are vast and currently do not allow for a randomised controlled trial. The current CoSMIC study is therefore limited to an observational, inception cohort study.

For peer review only

TRIAL REGISTRATION

National Research Ethics Service North West Committee (14/NW/1397). Trial registered at www.clinicaltrials.gov (NCT02456285).

KEYWORDS: Colorectal cancer; liver metastasis; synchronous; surgery; management.

For peer review only - http://bmjopen.bmj.com/site/about/guidelines.xhtml

BACKGROUND

Bowel cancer is the fourth most common cancer in the United Kingdom [1]. In Europe, colorectal cancer was the third most common cause of cancer and of cancer-related deaths in 2012 [2]. The liver is the most frequent site of metastasis in colorectal cancer: 14-20% of patients have hepatic metastases at presentation and up to a further third will subsequently develop liver lesions [3 4]. Liver metastases in patients with colorectal cancer are categorized as stage IV disease in which overall 5-year survival is 6% [5]. However, stage IV bowel cancer encompasses a wide clinical spectrum of disease ranging from patients with isolated hepatic metastases to patients with widespread metastatic disease. Patients with surgically resectable lesions confined to the liver have reported 5-year survival rates of 25 – 40% [6]. Such patients represent a selected but important sub-group in whom long-term survival of approximately 17% at 10-years is feasible when the hepatic metastatic burden is removed [7].

Patients who present with metastatic liver disease at a time point remote from their presentation with primary bowel cancer (termed metachronous disease) receive care focused on their new metastatic burden [8 9]. In contrast, the management of patients who present with colorectal cancer and concurrent liver metastases (termed synchronous metastases) are more complex [9 10]. These patients may have less favourable cancer biology and thus may be less likely to become long-term survivors [11]. Logically, the management of patients with colorectal cancer with synchronous metastases can be dichotomised into those with hepatic disease together with extra-hepatic metastatic disease and those with liver-limited metastatic disease. In the first category, systemic chemotherapy is the mainstay of treatment advocated in current guidelines for patients with advanced multi-site metastatic disease of colorectal cancer origin [9 12].

The second category of patients with liver-limited synchronous metastases represents a common and increasingly complex clinical management problem [13]. Traditional management (referred to variously as the classical or staged approach) comprised resection of the colorectal primary tumour followed by adjuvant chemotherapy with liver resection being undertaken (if at all) as a subsequent operation [13-15]. Key advances in the availability and safety of liver surgery, anaesthesia and critical care have made two alternative options feasible for patients with synchronous disease. The first is synchronous resection of the liver metastases and the

For peer review only

For peer review only - http://bmjopen.bmj.com/site/about/guidelines.xhtml

colorectal primary [13 15]. This has the attraction of removing the macroscopic tumour burden with a single operation. However, the morbidity of complex liver resection combined with major bowel resection may be considerable and there is some evidence of a negative effect on progression-free survival [16]. The second option is resection of the liver metastatic disease as the first step, termed the reverse or liver-first approach [17 18]. Liver-first surgery may be particularly applicable to patients with rectal cancer with synchronous liver metastases where pre-operative long-course chemo-radiotherapy for the rectal primary prior to surgical resection creates a potential "window" in which liver resection may be undertaken [19 20]. The liver-first strategy may also be oncologically advantageous if liver metastatic disease rather than the primary cancer gives rise to systemic metastasis – although this is not fully established [21]. A further potentially important benefit of the liver-first approach is that pelvic surgery may be either avoided or less extensive in patients with rectal tumours with a complete endoscopic, radiological and clinical response to chemo-radiotherapy [22].

Currently, evidence is inadequate to inform the selection of care pathway for patients with colorectal cancer with synchronous liver-limited hepatic metastases. Specifically, there is a dearth of prospectively recorded cohort-defining factors influencing treatment selection or outcome. European Society of Medical Oncology (ESMO) guidelines [9] provide only a framework for the management of these patients. In the United Kingdom's National Health Service (NHS) treatment decisions are made at multidisciplinary cancer team (MDT) meetings that include liver surgeons, colorectal surgeons, oncologists and specialist nurses. Factors considered in formulating a treatment pathway include co-morbidity and fitness for surgery, liver and colorectal disease distribution and the optimal placement of chemotherapy in the care plan.

Given the treatment permutations to be understood, an inception cohort study is valuable in order to understand patient outcomes as a function of clinical decisions and patient/disease characteristics. The CoSMIC inception cohort study will recruit patients with colorectal cancer with synchronous liver-limited hepatic metastatic disease, and aims to fulfil four objectives. First, the study will characterise the management of this cohort by reporting relationships between modes of presentation, management and adherence to or deviation from current clinical guidelines. The second objective of the CoSMIC study is to provide (for the first time) comparable outcome data on patients with colorectal cancer with liver-limited hepatic metastases treated by synchronous or sequential

For peer review only - http://bmjopen.bmj.com/site/about/guidelines.xhtml

surgery. The third objective is to address (also for the first time in a structured, prospective fashion) the impact of treatment on quality of life using validated questionnaire methodology. Finally, in this typically complex care plan it may be difficult for the patients' voice to be heard and given due consideration. The focus on patient experience is important [23], and may vary substantially according to the treatment pathway. Thus as a fourth objective a parallel qualitative study of both patient and clinician experience will help inform the knowledge of current practice. Completion of the CoSMIC study protocol will provide important new evidence about the treatment of patients with colorectal cancer with synchronous liver-limited metastases and provide objective evidence to guide future studies (including randomised evaluations) in this area.

For peer review only

For peer review only - http://bmjopen.bmj.com/site/about/guidelines.xhtml

METHODS AND ANALYSIS

Aims of the study

The primary aims of the study are to

1) Characterise the management of patients with colorectal cancer and synchronous liver metastases thus defining the relationship between presentation and treatment, in order to demonstrate adherence to or deviation from an evidence-informed common pathway. It is accepted that modern management of this complex clinical scenario cannot be sufficiently addressed by a single pathway but the guidelines suggested by ESMO provide constrained management options: these include early use of neo-adjuvant chemotherapy, surgical resection and adjuvant chemotherapy as the final stage. The treatment options within the common pathway standardise initial staging, accommodating treatment for liver metastases according to liver involvement and location of disease as well as different treatment requirements for patients with rectal primary cancer compared to those with colonic primary tumours

2) Provide comparable and prospective outcome data on patients with colorectal cancer with liver-limited hepatic metastases treated by synchronous or sequential surgery

3) To address the impact of treatment on quality of life using validated questionnaire methodology

4) To understand and explore the patient and care giver experience of their disease and their experiences through the treatment pathway, including their voice in treatment decision planning

5) To understand and explore the clinician's experience of providing care to patients, and their perspectives on treatment pathways, specifically in areas of clinical equipoise that make treatment allocation difficult

6) To explore the acceptability and barriers of a future randomized trial from both a clinician and patient's perspective, with a focus on the ethical dilemmas and the potential clinical value of such a study.

For peer review only - http://bmjopen.bmj.com/site/about/guidelines.xhtml

Design

An inception cohort study will evaluate the treatment and outcomes of patients with colorectal cancer with synchronous liver-limited hepatic metastases. A parallel phenomenological qualitative study will also explore the patient and care giver experience of the disease and treatment, and separately, the clinician perspective of providing care.

Setting

The study population will comprise patients with colorectal cancer with liver-limited hepatic metastases referred to the Hepatobiliary Surgical Unit at Manchester Royal Infirmary - an NHS regional cancer-network approved hepato-pancreato-biliary (HPB) centre with a formally constituted and National Cancer Network peer-review accredited MDT. The study opened for recruitment in April 2015 with prospective recruitment to be undertaken for 24 months.

Participants

In order to be eligible for inclusion in this cohort study, patients must fulfil the following:

Inclusion Criteria

1. Over 18 years of age.
2. Able to give informed consent.
3. Have a histological diagnosis of colorectal cancer.
4. No prior history of malignancy.

For peer review only - http://bmjopen.bmj.com/site/about/guidelines.xhtml

5. Have radiological evidence on either contrast-enhanced computed tomography (CT) or contrast-enhanced magnetic resonance (MR) scanning of hepatic metastases at the time of diagnosis of the primary tumour or within 3 months thereof. Liver metastases should not be biopsied.

6. CT and/or ^{18}fluoro-deoxyglucose positron emission tomographic (FDG-PET) evidence of the absence of extrahepatic metastases.

7. MR scan assessment of local stage in those patients with rectal primary tumours.

8. World Health Organisation performance status (PS) 0, 1 or 2 and considered by the MDT to be suitable for chemotherapy

9. A subset of patients from the cohort will be selected by purposeful sampling for the qualitative study following completion of their treatment, and are able to take part in a structured interview

Exclusion Criteria

1. Patients who are under 18 years of age.
2. Patients who are unable to give informed consent.
3. Patients who are unfit for the chemotherapy regimens in this protocol.
4. Any psychiatric or neurological condition assessed by clinical judgement to compromise the patient's ability to give informed consent or to comply with oral medication.
5. Partial or complete bowel obstruction not amenable to resolution by stent or diversion.
6. Pre-existing neuropathy (> grade 1).
7. Patients with another previous or current malignant disease.
8. Patients with known hypersensitivity reactions to any of the components of the study treatments.

For peer review only - http://bmjopen.bmj.com/site/about/guidelines.xhtml

9. Patients with distant metastases outwith the liver.

10. Patients who have received prior chemotherapy with oxaliplatin.

11. Patients with a personal or family history suggestive of dihydropyrimidine dehydrogenase (DPD) deficiency or with known DPD deficiency.

12. For patients selected for the qualitative study - those who are unable to give consent or are unfit to take part in a structured interview

For the clinician arm of the qualitative study, clinicians must fulfil the following:

Inclusion Criteria:

1. Consultant Grade

2. In clinical practice and an active participant of the HPB MDT in one of the following specialities: HPB Surgery, Colorectal Surgery, Radiology, Oncology and Histopathology

3. Willing to give informed consent

Exclusion Criteria:

1. Non-Consultant Grade

2. Not in clinical practice or an active participant of the HPB MDT

3. Unwilling to give informed consent

Recruitment

Patients will be formally identified prospectively at the weekly regional HPB MDT and approached in the outpatient clinic at MRI to discuss participation. Recruitment began in April 2015. For patients wishing to enrol,

For peer review only - http://bmjopen.bmj.com/site/about/guidelines.xhtml

but where the treatment pathway has already started (typically those who presented with an acute abdomen secondary to the bowel lesion), data will be retrospectively collected on treatment already received. Missing data points, particularly quality of life prior to surgery, during data analysis will be compensated for by unit imputation.

Potential participants of qualitative study will be approached following completion of their treatment either in the outpatient clinic or by telephone to ascertain their interest in taking part in the interviews.

Data collection

Clinical data will be collected on the following:

*Baseline staging investigations and treatment details (**Table One**)*

TABLE ONE

**Baseline Characteristics**
Patient Demographics
Charlson Co-Morbidity Score
Blood tests (FBC, serum urea and electrolytes, liver function tests, CEA)
**Clinical Presentation**
**Cancer Stage at Presentation**
Location and Stage (TNM/Dukes) of Colorectal Primary
Location, number and size of Liver Metastases
^{18}FDG-PET
Rectal MR (if applicable)
**Preoperative Workup**
Portal Vein Embolisation
CPET (cardiopulmonary exercise test)
**Surgery (Staged/Synchronous Resections)**
Sequence of Surgery
Open/Laparoscopic
Operative Time
Estimated Blood Loss / Transfusion
Bowel Resection

For peer review only - http://bmjopen.bmj.com/site/about/guidelines.xhtml

Primary Anastomosis
Covering Stoma
Liver Resection
Major Resection (> 3 Couinaud segments)
Pringle Time
Complications (Clavien-Dindo)
Critical Care Stay
Total Inpatient stay
Re-admission within 30 days
30-Day Mortality
**Chemotherapy (Neoadjuvant/Adjuvant)**
Regime
Number of Cycles (planned/given)
Duration of treatment
Side effects (Common Terminology Criteria for Adverse Events)
Restaging
**Outcomes (1, 2 and 5 years)**
Disease-free survival
Disease progression
**Quality of Life**
EQ-5D-3L
EORTC QLC-C30

In addition to demographic detail, baseline staging will include tests for histological confirmation of cancer such as biopsy-confirmation of a diagnosis of primary colorectal cancer (from the primary and not from the metastasis); tests for assessment of the liver and colorectal cancer in terms of lesion size, number, nodal involvement: contrast-enhanced CT scan and/or contrast-enhanced MR scan of the liver and pelvis and tests for assessment of the presence or absence of extra-hepatic metastatic disease such as ^{18}FDG-PET scan and serum assay of carcino-embryonic antigen (CEA). All of these tests are components of standard clinical care and no additional tests are undertaken for research purposes.

Predictors of treatment allocation

For peer review only - http://bmjopen.bmj.com/site/about/guidelines.xhtml

Factors which guide clinical decision-making in terms of the use of neoadjuvant chemotherapy and the choice of intervention (synchronous or sequential surgery).

Timelines for completion of the treatment protocol

For the purposes of this study, this is defined as the amount of time in days from enrolment to completion of the protocol. The term 'protocol' relates to completion of the common treatment pathway.

Failure to complete the treatment protocol

This is defined as drop-out prior to completion of the allocated treatment sequence. It will be further categorised as due to disease progression, patient choice or un-related to colorectal cancer (for example myocardial infarction) and will be recorded as the time in days from enrolment.

Disease-free survival 12 months after enrolment into protocol

This is defined as the absence of tumour on a CT scan of the thorax, abdomen and pelvis undertaken at the completion of the protocol. In the case of those patients with rectal tumours treated by a 'watch and wait' policy, the term disease-free can only be applied if there is a combination of radiological, endoscopic and clinical evidence of absence of cancer.

Disease progression in patients who are not disease-free at the end of protocol

The most sensitive measure of change is likely to involve a metric incorporating tumour size and number of lesions in the case of multiple metastases. There is evidence that CT-based volumetric assessment of metastases (seeded region growing method, slice-based segmentation or threshold-based segmentation) is more accurate for assessment of disease progression than the RECIST 1.1 method of largest axial diameter [24]. It is acknowledged that although RECIST criteria provide an objective means of assessment of solid tumour response to treatment, there is a risk of inter-observer bias [25]. Further, RECIST criteria may be insufficient to assess response to treatment in patients with colorectal liver metastases treated by biologic agents such as bevacizumab [26]. Thus, disease progression at end of protocol will be assessed both by RECIST 1.1 criteria and volumetric assessment.

For peer review only - http://bmjopen.bmj.com/site/about/guidelines.xhtml

Resection margin status

The terms R0 bowel resection and R0 liver resection will be used (R0 means no tumour at or within 1 mm of surgical resection margin) [27 28].

Complication and treatment-related morbidity profiles

Complications will be recorded prospectively according to the criteria defined above (see treatments) and assessed at the end of the study. Operative outcomes will be reported in keeping with the Dindo-Clavien system of assessment of post-operative morbidity [29]. The specific post-hepatectomy complications of haemorrhage [30], bile leakage [31] and liver failure [32] will be recorded in compliance with the guidance of the International Study Group of Liver Surgery. The morbidity associated with each intervention step will be recorded separately. Morbidity will include unplanned re-admission and re-operation. Requirement for non-elective surgery for colonic complications (obstruction, perforation, bleeding) will be recorded.

Mortality

Overall and cancer-related mortality in either arm after enrolment will be recorded. Mortality (and cause) will be determined using the Demographics Batch Service (DBS) to access the national electronic database of the UK NHS (National Health Service).

Use of stoma after colorectal surgery

Use of stoma (either temporary or permanent if this notification is available) will be recorded.

In-patient and critical care occupancy

A record will be made of in-patient and critical care occupancy associated with interventions; data will inform planning of economic evaluation in any subsequent randomised trial.

Quality of life

Quality of life (QoL) will be assessed using the European Organization for Research and Treatment of Cancer QLQ-LMC questionnaire, which has been validated for assessment of patient-reported outcomes during

For peer review only - http://bmjopen.bmj.com/site/about/guidelines.xhtml

treatment of colorectal liver metastases [33]. The questionnaire will be completed by patients at time of enrolment and at 12 and 24 months. The EuroQoL EQ5D-3L [34] will also be completed at the same time points, again supporting the design of future trial-based economic analyses.

Qualitative study interview guide

Structured interviews will last approximately 45 minutes, and explore (1) for patients and care givers: the experience of disease, particularly through the treatment pathway; understanding and expectations of timeframe for investigations; how they were informed of the diagnosis; how they received information related to the condition and treatment pathway; the type of information provided and who were the professionals explaining this; the nature and impact of information about diagnosis on patients and care givers, and on their relationship with the clinician; aspects of the process patients and care givers found useful/not so useful, and what could be improved; and the acceptability of entering into a future randomised control trial; (2) for clinicians: their experience of providing care, in particular, the perspectives and their views on treatment pathways; difficulties and challenges around treatment allocation and decision-making processes; the relationship with patients; acceptability and barriers to entering patients who may be under their care into a future randomized trial; any ethical issues and the potential clinical value of future randomized control study.

Data sources and measurements

Data will be collected prospectively using electronic study clinical case report forms. These will be anonymised and encrypted for storage and analysed prospectively during study to maximise data completion and resolve emergent problems in a timely fashion. The principal source of data will be the individual patient records. In addition, information will be gained by direct interview with patients (for quality of life assessment) and by interview with clinicians (for MDT choice decisions). Vital status beyond the duration of the study will be determined through the Demographics Batch Service of the NHS. Data will be reported at the end of year 3 allowing for a minimum 12 months outcome data in the entire cohort. It is also proposed (contingent on separate

For peer review only - http://bmjopen.bmj.com/site/about/guidelines.xhtml

funding) that information on outcome will be collected for up to 5 years from study commencement, providing an informative survival analysis of treatment options.

Qualitative interviews will be audio recorded and transcribed verbatim.

Study size

Based on clinical registers, the HPB unit at Manchester Royal Infirmary sees approximately 75 patients with colorectal cancer with synchronous liver-limited hepatic metastases per annum. As there are no study-related interventions, recruitment rates should be high and drop-out low and is estimated to provide 150 patients in the two-year recruitment period. A formal power calculation is not provided for this inception cohort study. Instead, the sample size is informed by the need to: provide stable estimates of variance for a range of outcomes; explore the relationship between the treatment pathway and health outcomes; estimate acceptability and recruitment rates; and describe patient and clinician experiences.

Purposeful sampling will be used to select patients from the cohort for the qualitative study. It is estimated that a sample size of 4-6 patients per group, and 1-2 clinicians will produce data saturation. However, we will continue to interview until data saturation is reached.

Analysis plan

The care of patients within the study pathway will be characterised by their principal treatment route as synchronous, liver-first or bowel-first. All patients will provide outcomes which will be included within analyses, grouped according to the treatment sequence received. Complication profiles in patients according to treatment group will be reported.

Statistical methods

For peer review only - http://bmjopen.bmj.com/site/about/guidelines.xhtml

Summary characteristics of patients, patient care provided and patient outcomes reported. Treatment centre characteristics will include measures of activity and surgical preference.

Exploratory analysis of process and clinical outcomes will be undertaken to explore the influence of patient, clinician, centre and treatment covariates, using regression modelling. Models will be subject to specification and robustness checks. Standard GLIM and propensity score matching (PSM) approaches will be compared to explore potential spectrum bias issues.

Interview transcripts will be managed by NVivo (QSR International Pty Ltd. Melborne, Australa) software. Interviews will be analysed thematically, using constant comparison [35] within a modified framework approach [36] coding both 'horizontally' (by coding each interview as a standalone hermeneutic unit) and 'vertically' (by scanning across the interviews for specific terms). Identified categories will be developed into a matrix of themes using mind-mapping techniques, and a systematic cross-comparison will be undertaken to identify the similarities and differences between the different types of participant.

Withdrawal from study

Patients will be able to withdraw from the study at any point. Data collected up to point of withdrawal will be retained for use within analyses.

Quality control measures

Colorectal cancer cases and patients with liver metastases will have their care discussed at an appropriately constituted, UK cancer-network approved MDT [37]. Quality control in radiological images. Cross-sectional imaging will comply with the recommendations for cross-sectional imaging in cancer management of the Royal College of Radiologists [38]. An independent Consultant Radiologist will head the radiology standards group. Quality control in histopathological reporting. All histopathology reporting will be in compliance with the guidelines of the Royal College of Pathologists [39].

For peer review only - http://bmjopen.bmj.com/site/about/guidelines.xhtml

Health Service cost of study

The clinical pathways within this study are cost neutral to the NHS as all the component steps are a part of current best-practice. The study provides a structured template for progression through this pathway but all components are currently best standard care. Currently and in the near future, scientific and clinical equipoise are likely to be maintained. It will be possible to explore determinants of resource use within the common pathway as a study outcome

Adverse event reporting

Adverse events will be recorded, assessed for severity and attribution, and reported in line with European Directive 2001/20/EC. In addition, if the Quality of Life assessment indicates that a patient is experiencing 'extreme problems' with their treatment, it would be an ethical duty of the CoSMIC research group to inform the clinical team involved with the care of the patient. This may introduce bias in subsequent quality of life assessments, and will be made transparent in any publication of results by the CoSMIC group.

Individual interviews will be stopped if there is any sign of emotional distress by either the patient or their relatives being interviewed. For any issues raised, with the patient's consent, we will contact their clinical team to make them aware of these issues so they can be formally addressed.

DISSEMINATION POLICY

The results of CoSMIC will be presented at the appropriate conferences. Study outcome data will be set at 1, 2 and 5 years. Following completion of publication of COSMIC findings, requests for study data will be considered, subject to meeting of institutional and data governance requirements.

For peer review only - http://bmjopen.bmj.com/site/about/guidelines.xhtml

ETHICS COMMITTEE APPROVALS.

The full study protocol was independently peer reviewed by Professor Kees de Jong (University of Maastricht, Holland). The CoSMIC study was approved by the National Research Ethics Service North West Committee (14/NW/1397) on the 9th November 2014 as well as local site ethics approval in each participating centre. The trial is registered at www.clinicaltrials.gov (NCT02456285).

For peer review only

For peer review only - http://bmjopen.bmj.com/site/about/guidelines.xhtml

ACKNOWLEDGEMENTS

* FULL LIST OF CoSMIC STUDY COLLABORATORS AND CO-AUTHORS

Aali J Sheen MD FRCS[1,2,3], Derek A O'Reilly PhD FRCS[1,2], Saurabh Jamdar MD FRCS[1,2], Rahul Deshpande MS FRCS[1], Nicola de Liguori Carino MD[1], Thomas Satyadas MS FRCS[1], Saifee Mullamitha MD MRCP[4], Michael Braun MRCP PhD[4], Nooreen Alam MRCP FRCR[4], Jurjees Hassan MSc MD FRCP[4], Gregory Wilson DRCOG FRCP Dip Onc[4], Thomas Treasure MD MS FRCS FRCP[5], Raj Rajashankar MRCP FRCR[6], Santhalingam Jegatheeswaran MRCS[1], Minas Baltatzis PhD[1], Professor Ray McMahon MD FRCPath[7], Rishi Sethi MRCS FRCR[6], James Hill ChM FRCS[8], David Smith BSc FRCS[9], Chris Smart FRCS[10], Arif Khan MS FRCS[11], Mohammud Kurrimboccus MD FRCS[12], Jonathan Epstein MD FRCS[13], Fergus Reid FRCS[14], Kamran Siddiqui FRCS[15], Ramesh Aswatha MD FRCS[16] and Marius Paraoan MD FRCS[17].

[1]Regional Hepato-Pancreato-Biliary Unit, Manchester Royal Infirmary, [2]Faculty of Medicine, University of Manchester, [3]Manchester Metropolitan University, [4]Oncology Service, Christie Hospital, Manchester, [5]Clinical Operation Research Unit, University College London, [6]Department of Radiology, Manchester Royal Infirmary, [7]Department of Histopathology, Manchester Royal Infirmary, [8]Colorectal Surgery Unit, Manchester Royal Infirmary, [9]Department of Surgery, Royal Bolton Hospital, [10]Department of Surgery, Macclesfield District General Hospital, [11]Department of Surgery, Leighton Hospital, Crewe, [12]Department of Surgery, North Manchester General Hospital, [13]Department of Surgery, Salford Royal Hospital, [14]Department of Surgery, Stepping Hill Hospital, [15]Department of Surgery, Tameside General Hospital, [16]Department of Surgery, Wythenshawe Hospital, [17]Department of Surgery, Royal Albert Edward Infirmary.

AUTHOR CONTRIBUTIONS

The study concept was conceived by Professor Ajith Siriwardena (AKS) and Professor James Mason (JM). All the authors - AKS, JM, Mr Anthony Chan (AC) and Dr Agnieszka Ignatowicz (AMI) - developed and modified the study design and protocol. AMI developed the qualitative aspect of the study. AC is currently undertaking the data collection and patient/clinician interviews, and the study forms part of a research PhD degree. All authors

For peer review only

will be involved in data analysis. AKS is the Chief Investigator of the study and takes overall responsibility for all aspects of the study design and trial conduct. All authors and collaborators have read and approved the final manuscript.

FUNDING STATEMENT

This work was supported by a grant from the Dickinson Trust of the Manchester Royal Infirmary (received by AC).

COMPETING INTERESTS

None.

TRIAL REGISTRATION

The study has been registered at www.clinical trials.gov (NCT02456285).

STUDY SPONSOR

CoSMIC is sponsored by Central Manchester University Hospitals NHS Foundation Trust,

For peer review only

For peer review only - http://bmjopen.bmj.com/site/about/guidelines.xhtml

REFERENCES

1. Cancer Research UK. Bowel (colorectal) cancer – UK incidence statistics. Secondary Cancer Research UK. Bowel (colorectal) cancer – UK incidence statistics. 2014

. http://info.cancerresearchuk.org/cancerstats/types/bowel/incidence/uk-bowel-cancer-incidence-statistics (accessed 15th January 2012).

2. Ferlay J, Steliarova-Foucher E, Lortet-Tieulent J, et al. Cancer incidence and mortality patterns in Europe: estimates for 40 countries in 2012. Eur J Cancer 2013;**49**(6):1374-403 doi: 10.1016/j.ejca.2012.12.027[published Online First: Epub Date]|.

3. Leporrier J, Maurel J, Chiche L, Bara S, Segol P, Launoy G. A population-based study of the incidence, management and prognosis of hepatic metastases from colorectal cancer. Br J Surg 2006;**93**(4):465-74 doi: 10.1002/bjs.5278[published Online First: Epub Date]|.

4. Manfredi S, Lepage C, Hatem C, Coatmeur O, Faivre J, Bouvier AM. Epidemiology and management of liver metastases from colorectal cancer. Ann Surg 2006;**244**(2):254-9 doi: 10.1097/01.sla.0000217629.94941.cf[published Online First: Epub Date]|.

5. Cancer Research UK. Bowel cancer –survival statistics. Secondary Cancer Research UK. Bowel cancer – survival statistics. 2014. http://info.cancerresearchuk.org/cancerstats/types/bowel/survival/ (accessed 15th January 2014).

6. Cancer Research UK, Statistics and outlook for bowel cancer Secondary Cancer Research UK, Statistics and outlook for bowel cancer 2014. http://www.cancerresearchuk.org/cancer-help/type/bowel-cancer/treatment/statistics-and-outlook-for-bowel-cancer (accessed 15hh January 2014).

7. Tomlinson JS, Jarnagin WR, DeMatteo RP, et al. Actual 10-year survival after resection of colorectal liver metastases defines cure. J Clin Oncol 2007;**25**(29):4575-80 doi: 10.1200/JCO.2007.11.0833[published Online First: Epub Date]|.

For peer review only - http://bmjopen.bmj.com/site/about/guidelines.xhtml

8. Jain VK, Hawkes EA, Cunningham D. Integration of biologic agents with cytotoxic chemotherapy in metastatic colorectal cancer. Clin Colorectal Cancer 2011;**10**(4):245-57 doi: 10.1016/j.clcc.2011.04.001[published Online First: Epub Date]|.

9. Schmoll HJ, Van Cutsem E, Stein A, et al. ESMO Consensus Guidelines for management of patients with colon and rectal cancer. a personalized approach to clinical decision making. Ann Oncol 2012;**23**(10):2479-516 doi: 10.1093/annonc/mds236[published Online First: Epub Date]|.

10. Bismuth H, Castaing D, Traynor O. Surgery for synchronous hepatic metastases of colorectal cancer. Scand J Gastroenterol Suppl 1988;**149**:144-9

11. Fong Y, Fortner J, Sun RL, Brennan MF, Blumgart LH. Clinical score for predicting recurrence after hepatic resection for metastatic colorectal cancer: analysis of 1001 consecutive cases. Ann Surg 1999;**230**(3):309-18; discussion 18-21

12. National Institute of Clinical Excellence. Colorectal Cancer: The diagnosis and management of colorectal cancer. Clinical Guideline CG 131, 2011.

13. Brouquet A, Mortenson MM, Vauthey JN, et al. Surgical strategies for synchronous colorectal liver metastases in 156 consecutive patients: classic, combined or reverse strategy? J Am Coll Surg 2010;**210**(6):934-41 doi: 10.1016/j.jamcollsurg.2010.02.039[published Online First: Epub Date]|.

14. Adam R, Lucidi V, Bismuth H. Hepatic colorectal metastases: methods of improving resectability. Surg Clin North Am 2004;**84**(2):659-71 doi: 10.1016/j.suc.2003.12.005[published Online First: Epub Date]|.

15. de Haas RJ, Adam R, Wicherts DA, et al. Comparison of simultaneous or delayed liver surgery for limited synchronous colorectal metastases. Br J Surg 2010;**97**(8):1279-89 doi: 10.1002/bjs.7106[published Online First: Epub Date]|.

16. Hillingso JG, Wille-Jorgensen P. Staged or simultaneous resection of synchronous liver metastases from colorectal cancer--a systematic review. Colorectal Dis 2009;**11**(1):3-10 doi: 10.1111/j.1463-1318.2008.01625.x[published Online First: Epub Date]|.

17. Mentha G, Majno PE, Andres A, Rubbia-Brandt L, Morel P, Roth AD. Neoadjuvant chemotherapy and resection of advanced synchronous liver metastases before treatment of the colorectal primary. Br J Surg 2006;**93**(7):872-8 doi: 10.1002/bjs.5346[published Online First: Epub Date]|.

For peer review only

For peer review only - http://bmjopen.bmj.com/site/about/guidelines.xhtml

18. Siriwardena AK, Mason JM, Mullamitha S, Hancock HC, Jegatheeswaran S. Management of colorectal cancer presenting with synchronous liver metastases. Nat Rev Clin Oncol 2014;**11**(8):446-59 doi: 10.1038/nrclinonc.2014.90[published Online First: Epub Date]|.

19. Aklilu M, Eng C. The current landscape of locally advanced rectal cancer. Nat Rev Clin Oncol 2011;**8**(11):649-59 doi: 10.1038/nrclinonc.2011.118[published Online First: Epub Date]|.

20. Sagar J. Colorectal stents for the management of malignant colonic obstructions. Cochrane Database Syst Rev 2011(11):CD007378 doi: 10.1002/14651858.CD007378.pub2[published Online First: Epub Date]|.

21. de Jong MC, van Dam RM, Maas M, et al. The liver-first approach for synchronous colorectal liver metastasis: a 5-year single-centre experience. HPB (Oxford) 2011;**13**(10):745-52 doi: 10.1111/j.1477-2574.2011.00372.x[published Online First: Epub Date]|.

22. O'Neill BD, Brown G, Heald RJ, Cunningham D, Tait DM. Non-operative treatment after neoadjuvant chemoradiotherapy for rectal cancer. Lancet Oncol 2007;**8**(7):625-33 doi: 10.1016/S1470-2045(07)70202-4[published Online First: Epub Date]|.

23. Department of Health. NHS Outcomes Framework 2016 to 2017. 2016

24. Fang WJ, Lam KO, Ng SC, et al. Manual contouring based volumetric evaluation for colorectal cancer with liver limited metastases: a comparison with RECIST. Asian Pac J Cancer Prev 2013;**14**(7):4151-5

25. Suzuki C, Torkzad MR, Jacobsson H, et al. Interobserver and intraobserver variability in the response evaluation of cancer therapy according to RECIST and WHO-criteria. Acta Oncol 2010;**49**(4):509-14 doi: 10.3109/02841861003705794[published Online First: Epub Date]|.

26. Lastoria S, Piccirillo MC, Caraco C, et al. Early PET/CT scan is more effective than RECIST in predicting outcome of patients with liver metastases from colorectal cancer treated with preoperative chemotherapy plus bevacizumab. J Nucl Med 2013;**54**(12):2062-9 doi: 10.2967/jnumed.113.119909[published Online First: Epub Date]|.

27. Vandeweyer D, Neo EL, Chen JW, Maddern GJ, Wilson TG, Padbury RT. Influence of resection margin on survival in hepatic resections for colorectal liver metastases. HPB (Oxford) 2009;**11**(6):499-504 doi: 10.1111/j.1477-2574.2009.00092.x[published Online First: Epub Date]|.

For peer review only - http://bmjopen.bmj.com/site/about/guidelines.xhtml

28. Edge S, Byrd D, Compton C, Fritz A, Greene F, Trotti A. *AJCC cancer staging manual*. 7th ed. New York: NY: Springer, 2010.

29. Dindo D, Demartines N, Clavien PA. Classification of surgical complications: a new proposal with evaluation in a cohort of 6336 patients and results of a survey. Ann Surg 2004;**240**(2):205-13

30. Rahbari NN, Garden OJ, Padbury R, et al. Post-hepatectomy haemorrhage: a definition and grading by the International Study Group of Liver Surgery (ISGLS). HPB (Oxford) 2011;**13**(8):528-35 doi: 10.1111/j.1477-2574.2011.00319.x[published Online First: Epub Date]|.

31. Koch M, Garden OJ, Padbury R, et al. Bile leakage after hepatobiliary and pancreatic surgery: a definition and grading of severity by the International Study Group of Liver Surgery. Surgery 2011;**149**(5):680-8 doi: 10.1016/j.surg.2010.12.002[published Online First: Epub Date]|.

32. Rahbari NN, Garden OJ, Padbury R, et al. Posthepatectomy liver failure: a definition and grading by the International Study Group of Liver Surgery (ISGLS). Surgery 2011;**149**(5):713-24 doi: 10.1016/j.surg.2010.10.001[published Online First: Epub Date]|.

33. Blazeby JM, Fayers P, Conroy T, et al. Validation of the European Organization for Research and Treatment of Cancer QLQ-LMC21 questionnaire for assessment of patient-reported outcomes during treatment of colorectal liver metastases. Br J Surg 2009;**96**(3):291-8 doi: 10.1002/bjs.6471[published Online First: Epub Date]|.

34. Szende A, Oppe M, Devlin N. *EQ-5D Value Sets: Inventory, Comparative Review and User Guide*: Springer Pub, 2007.

35. Glaser BG, Strauss AL. *The discovery of grounded theory: Strategies for qualitative research*: Aldine Publishing Company, 1967.

36. Richie J, Spencer L. *Qualitative data analysis for applied policy research*. London: Routledge, 1994.

37. Greater Manchester and Cheshire Cancer Network. Clinical sub-groups. Secondary Greater Manchester and Cheshire Cancer Network. Clinical sub-groups. 2012. http://www.gmccn.nhs.uk/hp/groups/clinicalSubGroups.

38. *The Royal College of Radiologists. Recommendations for cross-sectional imaging in cancer management.* London, 2006.

For peer review only - http://bmjopen.bmj.com/site/about/guidelines.xhtml

39. *The Royal College of Pathologists. Standards and datasets for reporting cancers. Dataset for colorectal cancer*. 2nd ed. London, 2007.

For peer review only

For peer review only - http://bmjopen.bmj.com/site/about/guidelines.xhtml

SPIRIT 2013 Checklist: Recommended items to address in a clinical trial protocol and related documents*

Section/item	Item No	Description
**Administrative information**		
Title	1	Descriptive title identifying the study design, population, interventions, and, if applicable, trial acronym **[Title Page (page 1)]**
Trial registration	2a	Trial identifier and registry name. If not yet registered, name of intended registry **[ClinicalTrials.gov (page 3)]**
	2b	All items from the World Health Organization Trial Registration Data Set **[All Items detailed in the study protocol]**
Protocol version	3	Date and version identifier **[version 1.0, date when published]**
Funding	4	Sources and types of financial, material, and other support **[Funding Statement (page 21)]**
Roles and responsibilities	5a	Names, affiliations, and roles of protocol contributors **[Names and Affiliations as stated on Title Page (page 1); Author Contributions (page 20)]**
	5b	Name and contact information for the trial sponsor **[Study Sponsor statement (page 21)]**
	5c	Role of study sponsor and funders, if any, in study design; collection, management, analysis, and interpretation of data; writing of the report; and the decision to submit the report for publication, including whether they will have ultimate authority over any of these activities **[Author Contributions (page 20)]**
	5d	Composition, roles, and responsibilities of the coordinating centre, steering committee, endpoint adjudication committee, data management team, and other individuals or groups overseeing the trial, if applicable (see Item 21a for data monitoring committee) **[Quality Control Measures (page 17)]**

For peer review only

Introduction

Background and rationale	6a	Description of research question and justification for undertaking the trial, including summary of relevant studies (published and unpublished) examining benefits and harms for each intervention **[Background (pages 4 - 6)]**
	6b	Explanation for choice of comparators **[Background (pages 4 - 6)]**
Objectives	7	Specific objectives or hypotheses **[Aims of the Study (page 7)]**
Trial design	8	Description of trial design including type of trial (eg, parallel group, crossover, factorial, single group), allocation ratio, and framework (eg, superiority, equivalence, noninferiority, exploratory) **[Design statement (page 8)]**

Methods: Participants, interventions, and outcomes

Study setting	9	Description of study settings (eg, community clinic, academic hospital) and list of countries where data will be collected. Reference to where list of study sites can be obtained **[Study Setting statement (page 8)]**
Eligibility criteria	10	Inclusion and exclusion criteria for participants. If applicable, eligibility criteria for study centres and individuals who will perform the interventions (eg, surgeons, psychotherapists) **[Inclusion and Exclusion Criteria (page 8 – 10)]**
Interventions	11a	Interventions for each group with sufficient detail to allow replication, including how and when they will be administered **[N/A]**
	11b	Criteria for discontinuing or modifying allocated interventions for a given trial participant (eg, drug dose change in response to harms, participant request, or improving/worsening disease) **[N/A]**
	11c	Strategies to improve adherence to intervention protocols, and any procedures for monitoring adherence (eg, drug tablet return, laboratory tests) **[N/A]**
	11d	Relevant concomitant care and interventions that are permitted or prohibited during the trial **[N/A]**

For peer review only - http://bmjopen.bmj.com/site/about/guidelines.xhtml

Outcomes	12	Primary, secondary, and other outcomes, including the specific measurement variable (eg, systolic blood pressure), analysis metric (eg, change from baseline, final value, time to event), method of aggregation (eg, median, proportion), and time point for each outcome. Explanation of the clinical relevance of chosen efficacy and harm outcomes is strongly recommended **[Data Collection (page 11 – 15)]**
Participant timeline	13	Time schedule of enrolment, interventions (including any run-ins and washouts), assessments, and visits for participants. A schematic diagram is highly recommended (see Figure) **[Recruitment (pages 10 - 11)]**
Sample size	14	Estimated number of participants needed to achieve study objectives and how it was determined, including clinical and statistical assumptions supporting any sample size calculations **[Study Size statement (page 16)]**
Recruitment	15	Strategies for achieving adequate participant enrolment to reach target sample size **[Recruitment (pages 10 - 11)]**
**Methods: Assignment of interventions (for controlled trials)**		
Allocation:		
Sequence generation	16a	Method of generating the allocation sequence (eg, computer-generated random numbers), and list of any factors for stratification. To reduce predictability of a random sequence, details of any planned restriction (eg, blocking) should be provided in a separate document that is unavailable to those who enrol participants or assign interventions **[N/A]**
Allocation concealment mechanism	16b	Mechanism of implementing the allocation sequence (eg, central telephone; sequentially numbered, opaque, sealed envelopes), describing any steps to conceal the sequence until interventions are assigned **[N/A]**
Implementation	16c	Who will generate the allocation sequence, who will enrol participants, and who will assign participants to interventions **[N/A]**
Blinding (masking)	17a	Who will be blinded after assignment to interventions (eg, trial participants, care providers, outcome assessors, data analysts), and how **[N/A]**

	17b	If blinded, circumstances under which unblinding is permissible, and procedure for revealing a participant's allocated intervention during the trial<br>**[N/A]**

Methods: Data collection, management, and analysis

Data collection methods	18a	Plans for assessment and collection of outcome, baseline, and other trial data, including any related processes to promote data quality (eg, duplicate measurements, training of assessors) and a description of study instruments (eg, questionnaires, laboratory tests) along with their reliability and validity, if known. Reference to where data collection forms can be found, if not in the protocol<br>**[Recruitment (pages 10-11) detailing collection of data, and Data Collection section (page 11 - 15) detailing data points]**
	18b	Plans to promote participant retention and complete follow-up, including list of any outcome data to be collected for participants who discontinue or deviate from intervention protocols<br>**[Withdrawal from study (page 17)]**
Data management	19	Plans for data entry, coding, security, and storage, including any related processes to promote data quality (eg, double data entry; range checks for data values). Reference to where details of data management procedures can be found, if not in the protocol<br>**[Data Sources and Measurements (pages 15 - 16)]**
Statistical methods	20a	Statistical methods for analysing primary and secondary outcomes. Reference to where other details of the statistical analysis plan can be found, if not in the protocol<br>**[Analysis Plan (page 16) and Statistical Methods (page 16 – 17)]**
	20b	Methods for any additional analyses (eg, subgroup and adjusted analyses)<br>**[Statistical Methods (page 16 – 17)]**
	20c	Definition of analysis population relating to protocol non-adherence (eg, as randomised analysis), and any statistical methods to handle missing data (eg, multiple imputation)<br>**[Statistical Methods (page 16 – 17)]**

Methods: Monitoring

Data monitoring	21a	Composition of data monitoring committee (DMC); summary of its role and reporting structure; statement of whether it is independent from the sponsor and competing interests; and reference to where further details about its charter can be found, if not in the protocol. Alternatively, an explanation of why a DMC is not needed<br>**[Quality Control Measures (page 17)]**

	21b	Description of any interim analyses and stopping guidelines, including who will have access to these interim results and make the final decision to terminate the trial **[N/A]**
Harms	22	Plans for collecting, assessing, reporting, and managing solicited and spontaneously reported adverse events and other unintended effects of trial interventions or trial conduct **[Adverse Events Reporting Statement (page 18)]**
Auditing	23	Frequency and procedures for auditing trial conduct, if any, and whether the process will be independent from investigators and the sponsor **[On-going audit from Sponsor's Research Department which is independent from the research team]**

Ethics and dissemination

Research ethics approval	24	Plans for seeking research ethics committee/institutional review board (REC/IRB) approval **[Ethics Committee Approval Statement (page 19)]**
Protocol amendments	25	Plans for communicating important protocol modifications (eg, changes to eligibility criteria, outcomes, analyses) to relevant parties (eg, investigators, REC/IRBs, trial participants, trial registries, journals, regulators) **[Any study ethics amendments will be communicated internally with all the investigators and collaborators. Study registration will be updated]**
Consent or assent	26a	Who will obtain informed consent or assent from potential trial participants or authorised surrogates, and how (see Item 32) **[Recruitment (pages 10 – 11)]**
	26b	Additional consent provisions for collection and use of participant data and biological specimens in ancillary studies, if applicable **[N/A]**
Confidentiality	27	How personal information about potential and enrolled participants will be collected, shared, and maintained in order to protect confidentiality before, during, and after the trial **[Data Sources and Measurements (page 15 – 16)]**
Declaration of interests	28	Financial and other competing interests for principal investigators for the overall trial and each study site **[Funding Statement and Competing Interests Statement (page 21)]**

For peer review only - http://bmjopen.bmj.com/site/about/guidelines.xhtml

Access to data	29	Statement of who will have access to the final trial dataset, and disclosure of contractual agreements that limit such access for investigators<br>**[Author Contributions (page 20) and Competing Interests (page 21)]**
Ancillary and post-trial care	30	Provisions, if any, for ancillary and post-trial care, and for compensation to those who suffer harm from trial participation<br>**[N/A]**
Dissemination policy	31a	Plans for investigators and sponsor to communicate trial results to participants, healthcare professionals, the public, and other relevant groups (eg, via publication, reporting in results databases, or other data sharing arrangements), including any publication restrictions<br>**[Dissemination Policy (Page 18)]**
	31b	Authorship eligibility guidelines and any intended use of professional writers<br>**[N/A]**
	31c	Plans, if any, for granting public access to the full protocol, participant-level dataset, and statistical code<br>**[Full protocol available online at ClinicalTrials.gov (details on page 21)]**
**Appendices**		
Informed consent materials	32	Model consent form and other related documentation given to participants and authorised surrogates<br>**[N/A]**
Biological specimens	33	Plans for collection, laboratory evaluation, and storage of biological specimens for genetic or molecular analysis in the current trial and for future use in ancillary studies, if applicable<br>**[N/A]**

*It is strongly recommended that this checklist be read in conjunction with the SPIRIT 2013 Explanation & Elaboration for important clarification on the items. Amendments to the protocol should be tracked and dated. The SPIRIT checklist is copyrighted by the SPIRIT Group under the Creative Commons "Attribution-NonCommercial-NoDerivs 3.0 Unported" license.

For peer review only

BMJ Open

Colorectal cancer with Synchronous liver-limited Metastases: The protocol of an Inception Cohort study (CoSMIC).

Journal:	*BMJ Open*
Manuscript ID	bmjopen-2016-015018.R1
Article Type:	Protocol
Date Submitted by the Author:	11-Jan-2017
Complete List of Authors:	Siriwardena, Ajith; Manchester Royal Infirmary, Manchester HepatoPancreatobiliary Centre<br>Chan, Anthony; Manchester Royal Infirmary, Department of Surgery<br>Ignatowicz, Agnieszka; Warwick Medical School, The University of Warwick, Division of Health Sciences<br>Mason, James; University of Warwick Warwick Medical School
**Primary Subject Heading**:	Surgery
Secondary Subject Heading:	Oncology
Keywords:	SURGERY, Colorectal surgery < SURGERY, Hepatobiliary surgery < SURGERY

SCHOLARONE™
Manuscripts

For peer review only

Revised re-submission bmjopen-2016-015018

Colorectal cancer with Synchronous liver-limited Metastases: The protocol of an Inception Cohort study (CoSMIC).

Ajith K Siriwardena MD FRCS[1,2], Anthony KC Chan MPhil MRCS[1,3], Agnieszka M Ignatowicz BSc MA PhD[3] and James M Mason DPhil[3] on behalf of the *CoSMIC study collaborators.

[1]Regional Hepato-Pancreato-Biliary Unit, Manchester Royal Infirmary, Oxford Road, Manchester, M13 9WL, UK

[2]Faculty of Medicine, University of Manchester, UK

[3]Warwick Medical School, University of Warwick, Coventry, CV4 7AL, UK

Correspondence to: Professor Ajith Siriwardena MD FRCS

Regional Hepato-Pancreato-Biliary Surgery Unit

Manchester Royal Infirmary

Oxford Road, Manchester M13 9WL.

Phone: +44 161 276 1234

Email: ajith.siriwardena@cmft.nhs.uk

Word Count (excluding title page, abstract, references, figures and tables): 4,072.

For peer review only

ABSTRACT

INTRODUCTION

Colorectal cancer is the fourth most common cancer in the UK and an important cause of cancer-related death. In 20% of patients, there is metastasis to the liver or beyond at the time of diagnosis. The management of synchronous disease is complex. Conventional surgery removes the colorectal primary first, followed by chemotherapy, with resection of liver metastases as a final step. Advances in the availability and safety of liver surgery, anaesthesia and critical care have made two alternative options feasible. The first is synchronous resection of the primary and liver metastases. The second is resection of the metastatic disease as the first step, termed the reverse or liver-first approach.

Currently, evidence is inadequate to inform the selection of care pathway for patients with colorectal cancer and synchronous liver-limited metastases. Specifically, optimal pathways are not defined and there is a dearth of prospectively recorded cohort-defining factors influencing treatment selection or outcome.

METHODS AND ANALYSIS

CoSMIC is an inception cohort study of patients with a new diagnosis of colorectal cancer with synchronous liver-limited metastases. The sequence of treatment received, and factors influencing treatment decisions, will be evaluated against European Society of Medical Oncology guidelines. Clinical data will be collected, and quality of life, morbidity, mortality and long-term outcome compared for different treatment sequences adjusted for prognostic factors. Disease-free survival or progression

For peer review only

For peer review only - http://bmjopen.bmj.com/site/about/guidelines.xhtml

will be measured at 1, 2 and 5 years. A nested qualitative study will ascertain patient experiences and clinician perspectives on delivery of care.

ETHICS AND DISSEMINATION

CoSMIC has ethical approval from the NHS Research Ethics Committee (14/NW/1397). Results will be disseminated to healthcare professionals and patient groups, and may be used to design a definitive trial addressing areas of equipoise in treatment pathways, as well as optimising current pathways to improve outcomes and experiences.

TRIAL REGISTRATION

ClinicalTrials.gov (NCT02456285).

KEYWORDS: Colorectal cancer; liver metastasis; synchronous; surgery; management.

For peer review only

For peer review only - http://bmjopen.bmj.com/site/about/guidelines.xhtml

STRENGTHS AND LIMITATIONS OF THIS STUDY

- First prospective study directly comparing outcomes between the different surgical sequences of patients with colorectal cancer and liver-limited metastases
- Completion of the CoSMIC study protocol will provide important new evidence about the treatment of patients with colorectal cancer with synchronous liver-limited metastases and provide objective evidence to guide future studies (including randomised evaluations) in this area.
- The variables involved in the treatment allocation of such patients are vast and currently do not allow for a randomised controlled trial. The current CoSMIC study is therefore limited to an observational, inception cohort study.

For peer review only - http://bmjopen.bmj.com/site/about/guidelines.xhtml

BACKGROUND

Bowel cancer is the fourth most common cancer in the United Kingdom [1]. In Europe, colorectal cancer was the third most common cause of cancer and of cancer-related deaths in 2012 [2]. The liver is the most frequent site of metastasis in colorectal cancer: 14-20% of patients have hepatic metastases at presentation and up to a further third will subsequently develop liver lesions [3 4]. Liver metastases in patients with colorectal cancer are categorized as stage IV disease in which overall 5-year survival is 6% [5]. However, stage IV bowel cancer encompasses a wide clinical spectrum of disease ranging from patients with isolated hepatic metastases to patients with widespread metastatic disease. Patients with surgically resectable lesions confined to the liver have reported 5-year survival rates of 25 – 40% [6]. Such patients represent a selected but important sub-group in whom long-term survival of approximately 17% at 10-years is feasible when the hepatic metastatic burden is removed [7].

Patients who present with metastatic liver disease at a time point remote from their presentation with primary bowel cancer (termed metachronous disease) receive care focused on their new metastatic burden [8 9]. In contrast, the management of patients who present with colorectal cancer and concurrent liver metastases (termed synchronous metastases) are more complex [9 10]. These patients may have less favourable cancer biology and thus may be less likely to become long-term survivors [11]. Logically, the management of patients with colorectal cancer with synchronous metastases can be dichotomised into those with hepatic disease together with extra-hepatic metastatic disease and those with liver-limited metastatic disease. In the first category, systemic chemotherapy is the mainstay of treatment advocated in current guidelines for patients with advanced multi-site metastatic disease of colorectal cancer origin [9 12].

The second category of patients with liver-limited synchronous metastases represents a common and increasingly complex clinical management problem [13]. Traditional management (referred to variously as the classical or staged approach) comprised resection of the colorectal primary tumour followed by adjuvant chemotherapy with liver resection being undertaken (if at all) as a subsequent operation [13-15]. Key advances in the availability and safety of liver surgery, anaesthesia and critical care have made two alternative options feasible for patients with synchronous disease. The first is synchronous resection of the liver metastases and the

For peer review only - http://bmjopen.bmj.com/site/about/guidelines.xhtml

colorectal primary [13 15]. This has the attraction of removing the macroscopic tumour burden with a single operation. However, the morbidity of complex liver resection combined with major bowel resection may be considerable and there is some evidence of a negative effect on progression-free survival [16]. The second option is resection of the liver metastatic disease as the first step, termed the reverse or liver-first approach [17 18]. Liver-first surgery may be particularly applicable to patients with rectal cancer with synchronous liver metastases where pre-operative long-course chemo-radiotherapy for the rectal primary prior to surgical resection creates a potential "window" in which liver resection may be undertaken [19 20]. The liver-first strategy may also be oncologically advantageous by addressing the hepatic metastatic burden before progression in the liver renders this unresectable [21]. A further potentially important benefit of the liver-first approach is that pelvic surgery may be either avoided or less extensive in patients with rectal tumours with a complete endoscopic, radiological and clinical response to chemo-radiotherapy [22].

Currently, evidence is inadequate to inform the selection of care pathway for patients with colorectal cancer with synchronous liver-limited hepatic metastases. Specifically, there is a dearth of prospectively recorded cohort-defining factors influencing treatment selection or outcome. European Society of Medical Oncology (ESMO) guidelines [9] provide only a framework for the management of these patients. In the United Kingdom's National Health Service (NHS) treatment decisions are made at multidisciplinary cancer team (MDT) meetings that include liver surgeons, colorectal surgeons, oncologists and specialist nurses. Factors considered in formulating a treatment pathway include co-morbidity and fitness for surgery, liver and colorectal disease distribution and the optimal placement of chemotherapy in the care plan.

Given the treatment permutations to be understood, an inception cohort study is valuable in order to understand patient outcomes as a function of clinical decisions and patient/disease characteristics. The CoSMIC inception cohort study will recruit patients with colorectal cancer with synchronous liver-limited hepatic metastatic disease, and aims to fulfil four objectives. First, the study will characterise the management of this cohort by reporting relationships between modes of presentation, management and adherence to or deviation from current clinical guidelines. The second objective of the CoSMIC study is to provide (for the first time) comparable outcome data on patients with colorectal cancer with liver-limited hepatic metastases treated by synchronous or sequential

For peer review only

For peer review only - http://bmjopen.bmj.com/site/about/guidelines.xhtml

surgery. The third objective is to address (also for the first time in a structured, prospective fashion) the impact of treatment on quality of life using validated questionnaire methodology. Finally, in this typically complex care plan it may be difficult for the patients' voice to be heard and given due consideration. The focus on patient experience is important [23], and may vary substantially according to the treatment pathway. Thus as a fourth objective a parallel qualitative study of both patient and clinician experience will help inform the knowledge of current practice. Completion of the CoSMIC study protocol will provide important new evidence about the treatment of patients with colorectal cancer with synchronous liver-limited metastases and provide objective evidence to guide future studies (including randomised evaluations) in this area.

For peer review only

For peer review only - http://bmjopen.bmj.com/site/about/guidelines.xhtml

METHODS AND ANALYSIS

Aims of the study

The primary aims of the study are to

1) Characterise the management of patients with colorectal cancer and synchronous liver metastases thus defining the relationship between presentation and treatment, in order to demonstrate adherence to or deviation from an evidence-informed common pathway. It is accepted that modern management of this complex clinical scenario cannot be sufficiently addressed by a single pathway but the guidelines suggested by ESMO provide constrained management options: these include early use of neo-adjuvant chemotherapy, surgical resection and adjuvant chemotherapy as the final stage. The treatment options within the common pathway standardise initial staging, accommodating treatment for liver metastases according to liver involvement and location of disease as well as different treatment requirements for patients with rectal primary cancer compared to those with colonic primary tumours

2) Provide comparable and prospective outcome data on patients with colorectal cancer with liver-limited hepatic metastases treated by synchronous or sequential surgery

3) To address the impact of treatment on quality of life using validated questionnaire methodology

4) To understand and explore the patient and care giver experience of their disease and their experiences through the treatment pathway, including their voice in treatment decision planning

5) To understand and explore the clinician's experience of providing care to patients, and their perspectives on treatment pathways, specifically in areas of clinical equipoise that make treatment allocation difficult

6) To explore the acceptability and barriers of a future randomized trial from both a clinician and patient's perspective, with a focus on the ethical dilemmas and the potential clinical value of such a study.

For peer review only - http://bmjopen.bmj.com/site/about/guidelines.xhtml

Design

An inception cohort study will evaluate the treatment and outcomes of patients with colorectal cancer with synchronous liver-limited hepatic metastases. A parallel phenomenological qualitative study will also explore the patient and care giver experience of the disease and treatment, and separately, the clinician perspective of providing care.

Setting

The study population will comprise patients with colorectal cancer with liver-limited hepatic metastases referred to the Hepatobiliary Surgical Unit at Manchester Royal Infirmary - an NHS regional cancer-network approved hepato-pancreato-biliary (HPB) centre with a formally constituted and National Cancer Network peer-review accredited MDT. The study opened for recruitment in April 2015 with prospective recruitment to be undertaken for 24 months.

Participants

In order to be eligible for inclusion in this cohort study, patients must fulfil the following:

Inclusion Criteria

1. Over 18 years of age.
2. Able to give informed consent.
3. Have a histological diagnosis of colorectal cancer.
4. No prior history of malignancy.

For peer review only - http://bmjopen.bmj.com/site/about/guidelines.xhtml

5. Have radiological evidence on either contrast-enhanced computed tomography (CT) or contrast-enhanced magnetic resonance (MR) scanning of hepatic metastases at the time of diagnosis of the primary tumour or within 3 months thereof. Liver metastases should not be biopsied.

6. CT and/or ^{18}fluoro-deoxyglucose positron emission tomographic (FDG-PET) evidence of the absence of extrahepatic metastases.

7. MR scan assessment of local stage in those patients with rectal primary tumours.

8. World Health Organisation performance status (PS) 0, 1 or 2 and considered by the MDT to be suitable for chemotherapy

9. A subset of patients from the cohort will be selected by purposeful sampling for the qualitative study following completion of their treatment, and are able to take part in a structured interview

Exclusion Criteria

1. Patients who are under 18 years of age.
2. Patients who are unable to give informed consent.
3. Patients who are unfit for the chemotherapy regimens in this protocol.
4. Any psychiatric or neurological condition assessed by clinical judgement to compromise the patient's ability to give informed consent or to comply with oral medication.
5. Partial or complete bowel obstruction not amenable to resolution by stent or diversion.
6. Pre-existing neuropathy (> grade 1).
7. Patients with another previous or current malignant disease.
8. Patients with known hypersensitivity reactions to any of the components of the study treatments.

For peer review only - http://bmjopen.bmj.com/site/about/guidelines.xhtml

9. Patients with distant metastases outwith the liver.
10. Patients who have received prior chemotherapy with oxaliplatin.
11. Patients with a personal or family history suggestive of dihydropyrimidine dehydrogenase (DPD) deficiency or with known DPD deficiency.
12. For patients selected for the qualitative study - those who are unable to give consent or are unfit to take part in a structured interview

For the clinician arm of the qualitative study, clinicians must fulfil the following:

Inclusion Criteria:

1. Consultant Grade
2. In clinical practice and an active participant of the HPB MDT in one of the following specialities: HPB Surgery, Colorectal Surgery, Radiology, Oncology and Histopathology
3. Willing to give informed consent

Exclusion Criteria:

1. Non-Consultant Grade
2. Not in clinical practice or an active participant of the HPB MDT
3. Unwilling to give informed consent

Recruitment

Patients will be formally identified prospectively at the weekly regional HPB MDT and approached in the outpatient clinic at MRI to discuss participation. Recruitment began in April 2015. For patients wishing to enrol,

For peer review only - http://bmjopen.bmj.com/site/about/guidelines.xhtml

but where the treatment pathway has already started (typically those who presented with an acute abdomen secondary to the bowel lesion), data will be retrospectively collected on treatment already received. Missing data points, particularly quality of life prior to surgery, during data analysis will be compensated for by unit imputation.

Potential participants of qualitative study will be approached following completion of their treatment either in the outpatient clinic or by telephone to ascertain their interest in taking part in the interviews.

Data collection

Clinical data will be collected on the following:

*Baseline staging investigations and treatment details (**Table One**)*

TABLE ONE

**Baseline Characteristics**
Patient Demographics
Charlson Co-Morbidity Score
Blood tests (FBC, serum urea and electrolytes, liver function tests, CEA)
**Clinical Presentation**
**Cancer Stage at Presentation**
Location and Stage (TNM/Dukes) of Colorectal Primary
Location, number and size of Liver Metastases
^{18}FDG-PET
Rectal MR (if applicable)
**Preoperative Workup**
Portal Vein Embolisation
CPET (cardiopulmonary exercise test)
**Surgery (Staged/Synchronous Resections)**
Sequence of Surgery
Open/Laparoscopic
Operative Time
Estimated Blood Loss / Transfusion
Bowel Resection

For peer review only - http://bmjopen.bmj.com/site/about/guidelines.xhtml

Primary Anastomosis	
Covering Stoma	
Liver Resection	
Major Resection (> 3 Couinaud segments)	
Pringle Time	
Complications (Clavien-Dindo)	
Critical Care Stay	
Total Inpatient stay	
Re-admission within 30 days	
30-Day Mortality	
**Chemotherapy (Neoadjuvant/Adjuvant)**	
Regime	
Number of Cycles (planned/given)	
Duration of treatment	
Side effects (Common Terminology Criteria for Adverse Events)	
Restaging	
**Outcomes (1, 2 and 5 years)**	
Disease-free survival	
Disease progression	
**Quality of Life**	
EQ-5D-3L	
EORTC QLC-C30	

In addition to demographic detail, baseline staging will include tests for histological confirmation of cancer such as biopsy-confirmation of a diagnosis of primary colorectal cancer (from the primary and not from the metastasis); tests for assessment of the liver and colorectal cancer in terms of lesion size, number, nodal involvement: contrast-enhanced CT scan and/or contrast-enhanced MR scan of the liver and pelvis and tests for assessment of the presence or absence of extra-hepatic metastatic disease such as ^{18}FDG-PET scan and serum assay of carcino-embryonic antigen (CEA). All of these tests are components of standard clinical care and no additional tests are undertaken for research purposes.

Predictors of treatment allocation

For peer review only - http://bmjopen.bmj.com/site/about/guidelines.xhtml

Factors which guide clinical decision-making in terms of the use of neoadjuvant chemotherapy and the choice of intervention (synchronous or sequential surgery).

Timelines for completion of the treatment protocol

For the purposes of this study, this is defined as the amount of time in days from enrolment to completion of the protocol. The term 'protocol' relates to completion of the common treatment pathway.

Failure to complete the treatment protocol

This is defined as drop-out prior to completion of the allocated treatment sequence. It will be further categorised as due to disease progression, patient choice or un-related to colorectal cancer (for example myocardial infarction) and will be recorded as the time in days from enrolment.

Disease-free survival 12 months after enrolment into protocol

This is defined as the absence of tumour on a CT scan of the thorax, abdomen and pelvis undertaken at the completion of the protocol. In the case of those patients with rectal tumours treated by a 'watch and wait' policy, the term disease-free can only be applied if there is a combination of radiological, endoscopic and clinical evidence of absence of cancer.

Disease progression in patients who are not disease-free at the end of protocol

The most sensitive measure of change is likely to involve a metric incorporating tumour size and number of lesions in the case of multiple metastases. There is evidence that CT-based volumetric assessment of metastases (seeded region growing method, slice-based segmentation or threshold-based segmentation) is more accurate for assessment of disease progression than the RECIST 1.1 method of largest axial diameter [24]. It is acknowledged that although RECIST criteria provide an objective means of assessment of solid tumour response to treatment, there is a risk of inter-observer bias [25]. Further, RECIST criteria may be insufficient to assess response to treatment in patients with colorectal liver metastases treated by biologic agents such as bevacizumab [26]. Thus, disease progression at end of protocol will be assessed both by RECIST 1.1 criteria and volumetric assessment.

For peer review only - http://bmjopen.bmj.com/site/about/guidelines.xhtml

Resection margin status

The terms R0 bowel resection and R0 liver resection will be used (R0 means no tumour at or within 1 mm of surgical resection margin) [27 28].

Complication and treatment-related morbidity profiles

Complications will be recorded prospectively according to the criteria defined above (see treatments) and assessed at the end of the study. Operative outcomes will be reported in keeping with the Dindo-Clavien system of assessment of post-operative morbidity [29]. The specific post-hepatectomy complications of haemorrhage [30], bile leakage [31] and liver failure [32] will be recorded in compliance with the guidance of the International Study Group of Liver Surgery. The morbidity associated with each intervention step will be recorded separately. Morbidity will include unplanned re-admission and re-operation. Requirement for non-elective surgery for colonic complications (obstruction, perforation, bleeding) will be recorded.

Mortality

Overall and cancer-related mortality in either arm after enrolment will be recorded. Mortality (and cause) will be determined using the Demographics Batch Service (DBS) to access the national electronic database of the UK NHS (National Health Service).

Use of stoma after colorectal surgery

Use of stoma (either temporary or permanent if this notification is available) will be recorded.

In-patient and critical care occupancy

A record will be made of in-patient and critical care occupancy associated with interventions; data will inform planning of economic evaluation in any subsequent randomised trial.

Quality of life

Quality of life (QoL) will be assessed using the European Organization for Research and Treatment of Cancer QLQ-LMC questionnaire, which has been validated for assessment of patient-reported outcomes during

For peer review only - http://bmjopen.bmj.com/site/about/guidelines.xhtml

treatment of colorectal liver metastases [33]. The questionnaire will be completed by patients at time of enrolment and at 12 and 24 months. The EuroQoL EQ5D-3L [34] will also be completed at the same time points, again supporting the design of future trial-based economic analyses.

Qualitative study interview guide

Structured interviews will last approximately 45 minutes, and explore (1) for patients and care givers: the experience of disease, particularly through the treatment pathway; understanding and expectations of timeframe for investigations; how they were informed of the diagnosis; how they received information related to the condition and treatment pathway; the type of information provided and who were the professionals explaining this; the nature and impact of information about diagnosis on patients and care givers, and on their relationship with the clinician; aspects of the process patients and care givers found useful/not so useful, and what could be improved; and the acceptability of entering into a future randomised control trial; (2) for clinicians: their experience of providing care, in particular, the perspectives and their views on treatment pathways; difficulties and challenges around treatment allocation and decision-making processes; the relationship with patients; acceptability and barriers to entering patients who may be under their care into a future randomized trial; any ethical issues and the potential clinical value of future randomized control study.

Data sources and measurements

Data will be collected prospectively using electronic study clinical case report forms. These will be anonymised and encrypted for storage and analysed prospectively during study to maximise data completion and resolve emergent problems in a timely fashion. The principal source of data will be the individual patient records. In addition, information will be gained by direct interview with patients (for quality of life assessment) and by interview with clinicians (for MDT choice decisions). Vital status beyond the duration of the study will be determined through the Demographics Batch Service of the NHS. Data will be reported at the end of year 3 allowing for a minimum 12 months outcome data in the entire cohort. It is also proposed (contingent on separate

For peer review only - http://bmjopen.bmj.com/site/about/guidelines.xhtml

funding) that information on outcome will be collected for up to 5 years from study commencement, providing an informative survival analysis of treatment options.

Qualitative interviews will be audio recorded and transcribed verbatim.

Study size

Based on clinical registers, the HPB unit at Manchester Royal Infirmary sees approximately 75 patients with colorectal cancer with synchronous liver-limited hepatic metastases per annum. As there are no study-related interventions, recruitment rates should be high and drop-out low and is estimated to provide 150 patients in the two-year recruitment period. A formal power calculation is not provided for this inception cohort study. Instead, the sample size is informed by the need to: provide stable estimates of variance for a range of outcomes; explore the relationship between the treatment pathway and health outcomes; estimate acceptability and recruitment rates; and describe patient and clinician experiences.

Purposeful sampling will be used to select patients from the cohort for the qualitative study. It is estimated that a sample size of 4-6 patients per group, and 1-2 clinicians will produce data saturation. However, we will continue to interview until data saturation is reached.

Analysis plan

The care of patients within the study pathway will be characterised by their principal treatment route as synchronous, liver-first or bowel-first. All patients will provide outcomes which will be included within analyses, grouped according to the treatment sequence received. Complication profiles in patients according to treatment group will be reported.

Acknowledgement of selection bias

For peer review only

For peer review only - http://bmjopen.bmj.com/site/about/guidelines.xhtml

The liver metastases multidisciplinary team meeting at the Manchester Royal Infirmary is the sole forum approved by cancer commissioners for discussion of the care of patients with colorectal cancer liver metastases. The HPB unit guideline is that all patient with stage IV colorectal cancer should have their care reviewed at the MDT. However, it is acknowledged that there are several groups of patients who may bypass the MDT. In particular, patients with systemic disease "beyond liver" may be referred for chemotherapy without consideration for liver surgery. From the patient's perspective, this care pathway is appropriate. Similarly, patients who present to local MDTs with liver metastases who undergo bowel-first surgery but whose disease progresses rendering them unsuitable for consideration for liver surgery will likely not be referred. For the purposes of reporting the CoSMIC data these sources of patient loss to study will be acknowledged together with any potential for selection bias. Reporting will be pragmatic and descriptive.

Statistical methods

Summary characteristics of patients, patient care provided and patient outcomes reported. Treatment centre characteristics will include measures of activity and surgical preference.

Exploratory analysis of process and clinical outcomes will be undertaken to explore the influence of patient, clinician, centre and treatment covariates, using regression modelling. Models will be subject to specification and robustness checks. Standard GLIM and propensity score matching (PSM) approaches will be compared to explore potential spectrum bias issues.

Interview transcripts will be managed by NVivo (QSR International Pty Ltd. Melborne, Australa) software. Interviews will be analysed thematically, using constant comparison [35] within a modified framework approach [36] coding both 'horizontally' (by coding each interview as a standalone hermeneutic unit) and 'vertically' (by scanning across the interviews for specific terms). Identified categories will be developed into a matrix of themes using mind-mapping techniques, and a systematic cross-comparison will be undertaken to identify the similarities and differences between the different types of participant.

For peer review only - http://bmjopen.bmj.com/site/about/guidelines.xhtml

Withdrawal from study

Patients will be able to withdraw from the study at any point. Data collected up to point of withdrawal will be retained for use within analyses.

Quality control measures

Colorectal cancer cases and patients with liver metastases will have their care discussed at an appropriately constituted, UK cancer-network approved MDT [37]. Quality control in radiological images. Cross-sectional imaging will comply with the recommendations for cross-sectional imaging in cancer management of the Royal College of Radiologists [38]. An independent Consultant Radiologist will head the radiology standards group. Quality control in histopathological reporting. All histopathology reporting will be in compliance with the guidelines of the Royal College of Pathologists [39].

Health Service cost of study

The clinical pathways within this study are cost neutral to the NHS as all the component steps are a part of current best-practice. The study provides a structured template for progression through this pathway but all components are currently best standard care. Currently and in the near future, scientific and clinical equipoise are likely to be maintained. It will be possible to explore determinants of resource use within the common pathway as a study outcome

Adverse event reporting

Adverse events will be recorded, assessed for severity and attribution, and reported in line with European Directive 2001/20/EC. In addition, if the Quality of Life assessment indicates that a patient is experiencing 'extreme problems' with their treatment, it would be an ethical duty of the CoSMIC research group to inform the clinical team involved with the care of the patient. This may introduce bias in subsequent quality of life assessments, and will be made transparent in any publication of results by the CoSMIC group.

For peer review only - http://bmjopen.bmj.com/site/about/guidelines.xhtml

Individual interviews will be stopped if there is any sign of emotional distress by either the patient or their relatives being interviewed. For any issues raised, with the patient's consent, we will contact their clinical team to make them aware of these issues so they can be formally addressed.

DISSEMINATION POLICY

The results of CoSMIC will be presented at the appropriate conferences. Study outcome data will be set at 1, 2 and 5 years. Following publication of the final results, anonymised raw data will be made available.

For peer review only

For peer review only - http://bmjopen.bmj.com/site/about/guidelines.xhtml

ETHICS COMMITTEE APPROVALS.

The full study protocol was independently peer reviewed by Professor Kees de Jong (University of Maastricht, Holland). The CoSMIC study was approved by the National Research Ethics Service North West Committee (14/NW/1397) on the 9th November 2014 as well as local site ethics approval in each participating centre. The trial is registered at www.clinicaltrials.gov (NCT02456285).

For peer review only

For peer review only - http://bmjopen.bmj.com/site/about/guidelines.xhtml

ACKNOWLEDGEMENTS

* FULL LIST OF CoSMIC STUDY COLLABORATORS AND CO-AUTHORS

Aali J Sheen MD FRCS[1,2,3], Derek A O'Reilly PhD FRCS[1,2], Saurabh Jamdar MD FRCS[1,2], Rahul Deshpande MS FRCS[1], Nicola de Liguori Carino MD[1], Thomas Satyadas MS FRCS[1], Saifee Mullamitha MD MRCP[4], Michael Braun MRCP PhD[4], Nooreen Alam MRCP FRCR[4], Jurjees Hassan MSc MD FRCP[4], Gregory Wilson DRCOG FRCP Dip Onc[4], Thomas Treasure MD MS FRCS FRCP[5], Raj Rajashankar MRCP FRCR[6], Santhalingam Jegatheeswaran MRCS[1], Minas Baltatzis PhD[1], Professor Ray McMahon MD FRCPath[7], Rishi Sethi MRCS FRCR[6], James Hill ChM FRCS[8], David Smith BSc FRCS[9], Chris Smart FRCS[10], Arif Khan MS FRCS[11], Mohammud Kurrimboccus MD FRCS[12], Jonathan Epstein MD FRCS[13], Fergus Reid FRCS[14], Kamran Siddiqui FRCS[15], Ramesh Aswatha MD FRCS[16] and Marius Paraoan MD FRCS[17].

[1]Regional Hepato-Pancreato-Biliary Unit, Manchester Royal Infirmary, [2]Faculty of Medicine, University of Manchester, [3]Manchester Metropolitan University, [4]Oncology Service, Christie Hospital, Manchester, [5]Clinical Operation Research Unit, University College London, [6]Department of Radiology, Manchester Royal Infirmary, [7]Department of Histopathology, Manchester Royal Infirmary, [8]Colorectal Surgery Unit, Manchester Royal Infirmary, [9]Department of Surgery, Royal Bolton Hospital, [10]Department of Surgery, Macclesfield District General Hospital, [11]Department of Surgery, Leighton Hospital, Crewe, [12]Department of Surgery, North Manchester General Hospital, [13]Department of Surgery, Salford Royal Hospital, [14]Department of Surgery, Stepping Hill Hospital, [15]Department of Surgery, Tameside General Hospital, [16]Department of Surgery, Wythenshawe Hospital, [17]Department of Surgery, Royal Albert Edward Infirmary.

AUTHOR CONTRIBUTIONS

AKS and JM conceived the study concept. All the authors developed and modified the study design and protocol. AKS is the Chief Investigator of the study and takes overall responsibility for all aspects of the study design and trial conduct. All authors and collaborators have read and approved the final manuscript.

For peer review only

For peer review only - http://bmjopen.bmj.com/site/about/guidelines.xhtml

FUNDING STATEMENT

This work was supported by a grant from the Dickinson Trust of the Manchester Royal Infirmary (received by AC).

COMPETING INTERESTS

None.

TRIAL REGISTRATION

The study has been registered at www.clinical trials.gov (NCT02456285).

STUDY SPONSOR

CoSMIC is sponsored by Central Manchester University Hospitals NHS Foundation Trust,

For peer review only

REFERENCES

1. Cancer Research UK. Bowel (colorectal) cancer – UK incidence statistics. Secondary Cancer Research UK. Bowel (colorectal) cancer – UK incidence statistics. 2014

. http://info.cancerresearchuk.org/cancerstats/types/bowel/incidence/uk-bowel-cancer-incidence-statistics (accessed 15th January 2012).

2. Ferlay J, Steliarova-Foucher E, Lortet-Tieulent J, et al. Cancer incidence and mortality patterns in Europe: estimates for 40 countries in 2012. Eur J Cancer 2013;**49**(6):1374-403 doi: 10.1016/j.ejca.2012.12.027[published Online First: Epub Date]|.

3. Leporrier J, Maurel J, Chiche L, Bara S, Segol P, Launoy G. A population-based study of the incidence, management and prognosis of hepatic metastases from colorectal cancer. Br J Surg 2006;**93**(4):465-74 doi: 10.1002/bjs.5278[published Online First: Epub Date]|.

4. Manfredi S, Lepage C, Hatem C, Coatmeur O, Faivre J, Bouvier AM. Epidemiology and management of liver metastases from colorectal cancer. Ann Surg 2006;**244**(2):254-9 doi: 10.1097/01.sla.0000217629.94941.cf[published Online First: Epub Date]|.

5. Cancer Research UK. Bowel cancer –survival statistics. Secondary Cancer Research UK. Bowel cancer – survival statistics. 2014. http://info.cancerresearchuk.org/cancerstats/types/bowel/survival/ (accessed 15th January 2014).

6. Cancer Research UK, Statistics and outlook for bowel cancer Secondary Cancer Research UK, Statistics and outlook for bowel cancer 2014. http://www.cancerresearchuk.org/cancer-help/type/bowel-cancer/treatment/statistics-and-outlook-for-bowel-cancer (accessed 15hh January 2014).

7. Tomlinson JS, Jarnagin WR, DeMatteo RP, et al. Actual 10-year survival after resection of colorectal liver metastases defines cure. J Clin Oncol 2007;**25**(29):4575-80 doi: 10.1200/JCO.2007.11.0833[published Online First: Epub Date]|.

For peer review only - http://bmjopen.bmj.com/site/about/guidelines.xhtml

8. Jain VK, Hawkes EA, Cunningham D. Integration of biologic agents with cytotoxic chemotherapy in metastatic colorectal cancer. Clin Colorectal Cancer 2011;**10**(4):245-57 doi: 10.1016/j.clcc.2011.04.001[published Online First: Epub Date]|.

9. Schmoll HJ, Van Cutsem E, Stein A, et al. ESMO Consensus Guidelines for management of patients with colon and rectal cancer. a personalized approach to clinical decision making. Ann Oncol 2012;**23**(10):2479-516 doi: 10.1093/annonc/mds236[published Online First: Epub Date]|.

10. Bismuth H, Castaing D, Traynor O. Surgery for synchronous hepatic metastases of colorectal cancer. Scand J Gastroenterol Suppl 1988;**149**:144-9

11. Fong Y, Fortner J, Sun RL, Brennan MF, Blumgart LH. Clinical score for predicting recurrence after hepatic resection for metastatic colorectal cancer: analysis of 1001 consecutive cases. Ann Surg 1999;**230**(3):309-18; discussion 18-21

12. National Institute of Clinical Excellence. Colorectal Cancer: The diagnosis and management of colorectal cancer. Clinical Guideline CG 131, 2011.

13. Brouquet A, Mortenson MM, Vauthey JN, et al. Surgical strategies for synchronous colorectal liver metastases in 156 consecutive patients: classic, combined or reverse strategy? J Am Coll Surg 2010;**210**(6):934-41 doi: 10.1016/j.jamcollsurg.2010.02.039[published Online First: Epub Date]|.

14. Adam R, Lucidi V, Bismuth H. Hepatic colorectal metastases: methods of improving resectability. Surg Clin North Am 2004;**84**(2):659-71 doi: 10.1016/j.suc.2003.12.005[published Online First: Epub Date]|.

15. de Haas RJ, Adam R, Wicherts DA, et al. Comparison of simultaneous or delayed liver surgery for limited synchronous colorectal metastases. Br J Surg 2010;**97**(8):1279-89 doi: 10.1002/bjs.7106[published Online First: Epub Date]|.

16. Hillingso JG, Wille-Jorgensen P. Staged or simultaneous resection of synchronous liver metastases from colorectal cancer--a systematic review. Colorectal Dis 2009;**11**(1):3-10 doi: 10.1111/j.1463-1318.2008.01625.x[published Online First: Epub Date]|.

17. Mentha G, Majno PE, Andres A, Rubbia-Brandt L, Morel P, Roth AD. Neoadjuvant chemotherapy and resection of advanced synchronous liver metastases before treatment of the colorectal primary. Br J Surg 2006;**93**(7):872-8 doi: 10.1002/bjs.5346[published Online First: Epub Date]|.

For peer review only - http://bmjopen.bmj.com/site/about/guidelines.xhtml

18. Siriwardena AK, Mason JM, Mullamitha S, Hancock HC, Jegatheeswaran S. Management of colorectal cancer presenting with synchronous liver metastases. Nat Rev Clin Oncol 2014;**11**(8):446-59 doi: 10.1038/nrclinonc.2014.90[published Online First: Epub Date]|.

19. Aklilu M, Eng C. The current landscape of locally advanced rectal cancer. Nat Rev Clin Oncol 2011;**8**(11):649-59 doi: 10.1038/nrclinonc.2011.118[published Online First: Epub Date]|.

20. Sagar J. Colorectal stents for the management of malignant colonic obstructions. Cochrane Database Syst Rev 2011(11):CD007378 doi: 10.1002/14651858.CD007378.pub2[published Online First: Epub Date]|.

21. de Jong MC, van Dam RM, Maas M, et al. The liver-first approach for synchronous colorectal liver metastasis: a 5-year single-centre experience. HPB (Oxford) 2011;**13**(10):745-52 doi: 10.1111/j.1477-2574.2011.00372.x[published Online First: Epub Date]|.

22. O'Neill BD, Brown G, Heald RJ, Cunningham D, Tait DM. Non-operative treatment after neoadjuvant chemoradiotherapy for rectal cancer. Lancet Oncol 2007;**8**(7):625-33 doi: 10.1016/S1470-2045(07)70202-4[published Online First: Epub Date]|.

23. Department of Health. NHS Outcomes Framework 2016 to 2017. 2016

24. Fang WJ, Lam KO, Ng SC, et al. Manual contouring based volumetric evaluation for colorectal cancer with liver limited metastases: a comparison with RECIST. Asian Pac J Cancer Prev 2013;**14**(7):4151-5

25. Suzuki C, Torkzad MR, Jacobsson H, et al. Interobserver and intraobserver variability in the response evaluation of cancer therapy according to RECIST and WHO-criteria. Acta Oncol 2010;**49**(4):509-14 doi: 10.3109/02841861003705794[published Online First: Epub Date]|.

26. Lastoria S, Piccirillo MC, Caraco C, et al. Early PET/CT scan is more effective than RECIST in predicting outcome of patients with liver metastases from colorectal cancer treated with preoperative chemotherapy plus bevacizumab. J Nucl Med 2013;**54**(12):2062-9 doi: 10.2967/jnumed.113.119909[published Online First: Epub Date]|.

27. Vandeweyer D, Neo EL, Chen JW, Maddern GJ, Wilson TG, Padbury RT. Influence of resection margin on survival in hepatic resections for colorectal liver metastases. HPB (Oxford) 2009;**11**(6):499-504 doi: 10.1111/j.1477-2574.2009.00092.x[published Online First: Epub Date]|.

For peer review only - http://bmjopen.bmj.com/site/about/guidelines.xhtml

28. Edge S, Byrd D, Compton C, Fritz A, Greene F, Trotti A. *AJCC cancer staging manual*. 7th ed. New York: NY: Springer, 2010.

29. Dindo D, Demartines N, Clavien PA. Classification of surgical complications: a new proposal with evaluation in a cohort of 6336 patients and results of a survey. Ann Surg 2004;**240**(2):205-13

30. Rahbari NN, Garden OJ, Padbury R, et al. Post-hepatectomy haemorrhage: a definition and grading by the International Study Group of Liver Surgery (ISGLS). HPB (Oxford) 2011;**13**(8):528-35 doi: 10.1111/j.1477-2574.2011.00319.x[published Online First: Epub Date]|.

31. Koch M, Garden OJ, Padbury R, et al. Bile leakage after hepatobiliary and pancreatic surgery: a definition and grading of severity by the International Study Group of Liver Surgery. Surgery 2011;**149**(5):680-8 doi: 10.1016/j.surg.2010.12.002[published Online First: Epub Date]|.

32. Rahbari NN, Garden OJ, Padbury R, et al. Posthepatectomy liver failure: a definition and grading by the International Study Group of Liver Surgery (ISGLS). Surgery 2011;**149**(5):713-24 doi: 10.1016/j.surg.2010.10.001[published Online First: Epub Date]|.

33. Blazeby JM, Fayers P, Conroy T, et al. Validation of the European Organization for Research and Treatment of Cancer QLQ-LMC21 questionnaire for assessment of patient-reported outcomes during treatment of colorectal liver metastases. Br J Surg 2009;**96**(3):291-8 doi: 10.1002/bjs.6471[published Online First: Epub Date]|.

34. Szende A, Oppe M, Devlin N. *EQ-5D Value Sets: Inventory, Comparative Review and User Guide*: Springer Pub, 2007.

35. Glaser BG, Strauss AL. *The discovery of grounded theory: Strategies for qualitative research*: Aldine Publishing Company, 1967.

36. Richie J, Spencer L. *Qualitative data analysis for applied policy research*. London: Routledge, 1994.

37. Greater Manchester and Cheshire Cancer Network. Clinical sub-groups. Secondary Greater Manchester and Cheshire Cancer Network. Clinical sub-groups. 2012. http://www.gmccn.nhs.uk/hp/groups/clinicalSubGroups.

38. *The Royal College of Radiologists. Recommendations for cross-sectional imaging in cancer management*. London, 2006.

For peer review only - http://bmjopen.bmj.com/site/about/guidelines.xhtml

39. *The Royal College of Pathologists. Standards and datasets for reporting cancers. Dataset for colorectal cancer*. 2nd ed. London, 2007.

For peer review only

SPIRIT
STANDARD PROTOCOL ITEMS: RECOMMENDATIONS FOR INTERVENTIONAL TRIALS

SPIRIT 2013 Checklist: Recommended items to address in a clinical trial protocol and related documents*

Section/item	Item No	Description
**Administrative information**		
Title	1	Descriptive title identifying the study design, population, interventions, and, if applicable, trial acronym **[Title Page (page 1)]**
Trial registration	2a	Trial identifier and registry name. If not yet registered, name of intended registry **[ClinicalTrials.gov (page 3)]**
	2b	All items from the World Health Organization Trial Registration Data Set **[All Items detailed in the study protocol]**
Protocol version	3	Date and version identifier **[version 1.0, date when published]**
Funding	4	Sources and types of financial, material, and other support **[Funding Statement (page 21)]**
Roles and responsibilities	5a	Names, affiliations, and roles of protocol contributors **[Names and Affiliations as stated on Title Page (page 1); Author Contributions (page 20)]**
	5b	Name and contact information for the trial sponsor **[Study Sponsor statement (page 21)]**
	5c	Role of study sponsor and funders, if any, in study design; collection, management, analysis, and interpretation of data; writing of the report; and the decision to submit the report for publication, including whether they will have ultimate authority over any of these activities **[Author Contributions (page 20)]**
	5d	Composition, roles, and responsibilities of the coordinating centre, steering committee, endpoint adjudication committee, data management team, and other individuals or groups overseeing the trial, if applicable (see Item 21a for data monitoring committee) **[Quality Control Measures (page 17)]**

For peer review only

Introduction

Background and rationale	6a	Description of research question and justification for undertaking the trial, including summary of relevant studies (published and unpublished) examining benefits and harms for each intervention **[Background (pages 4 - 6)]**
	6b	Explanation for choice of comparators **[Background (pages 4 - 6)]**
Objectives	7	Specific objectives or hypotheses **[Aims of the Study (page 7)]**
Trial design	8	Description of trial design including type of trial (eg, parallel group, crossover, factorial, single group), allocation ratio, and framework (eg, superiority, equivalence, noninferiority, exploratory) **[Design statement (page 8)]**

Methods: Participants, interventions, and outcomes

Study setting	9	Description of study settings (eg, community clinic, academic hospital) and list of countries where data will be collected. Reference to where list of study sites can be obtained **[Study Setting statement (page 8)]**
Eligibility criteria	10	Inclusion and exclusion criteria for participants. If applicable, eligibility criteria for study centres and individuals who will perform the interventions (eg, surgeons, psychotherapists) **[Inclusion and Exclusion Criteria (page 8 – 10)]**
Interventions	11a	Interventions for each group with sufficient detail to allow replication, including how and when they will be administered **[N/A]**
	11b	Criteria for discontinuing or modifying allocated interventions for a given trial participant (eg, drug dose change in response to harms, participant request, or improving/worsening disease) **[N/A]**
	11c	Strategies to improve adherence to intervention protocols, and any procedures for monitoring adherence (eg, drug tablet return, laboratory tests) **[N/A]**
	11d	Relevant concomitant care and interventions that are permitted or prohibited during the trial **[N/A]**

For peer review only - http://bmjopen.bmj.com/site/about/guidelines.xhtml

Outcomes	12	Primary, secondary, and other outcomes, including the specific measurement variable (eg, systolic blood pressure), analysis metric (eg, change from baseline, final value, time to event), method of aggregation (eg, median, proportion), and time point for each outcome. Explanation of the clinical relevance of chosen efficacy and harm outcomes is strongly recommended<br>**[Data Collection (page 11 – 15)]**
Participant timeline	13	Time schedule of enrolment, interventions (including any run-ins and washouts), assessments, and visits for participants. A schematic diagram is highly recommended (see Figure)<br>**[Recruitment (pages 10 - 11)]**
Sample size	14	Estimated number of participants needed to achieve study objectives and how it was determined, including clinical and statistical assumptions supporting any sample size calculations<br>**[Study Size statement (page 16)]**
Recruitment	15	Strategies for achieving adequate participant enrolment to reach target sample size<br>**[Recruitment (pages 10 - 11)]**

Methods: Assignment of interventions (for controlled trials)

Allocation:

Sequence generation	16a	Method of generating the allocation sequence (eg, computer-generated random numbers), and list of any factors for stratification. To reduce predictability of a random sequence, details of any planned restriction (eg, blocking) should be provided in a separate document that is unavailable to those who enrol participants or assign interventions<br>**[N/A]**
Allocation concealment mechanism	16b	Mechanism of implementing the allocation sequence (eg, central telephone; sequentially numbered, opaque, sealed envelopes), describing any steps to conceal the sequence until interventions are assigned<br>**[N/A]**
Implementation	16c	Who will generate the allocation sequence, who will enrol participants, and who will assign participants to interventions<br>**[N/A]**
Blinding (masking)	17a	Who will be blinded after assignment to interventions (eg, trial participants, care providers, outcome assessors, data analysts), and how<br>**[N/A]**

	17b	If blinded, circumstances under which unblinding is permissible, and procedure for revealing a participant's allocated intervention during the trial<br>**[N/A]**
**Methods: Data collection, management, and analysis**		
Data collection methods	18a	Plans for assessment and collection of outcome, baseline, and other trial data, including any related processes to promote data quality (eg, duplicate measurements, training of assessors) and a description of study instruments (eg, questionnaires, laboratory tests) along with their reliability and validity, if known. Reference to where data collection forms can be found, if not in the protocol<br>**[Recruitment (pages 10-11) detailing collection of data, and Data Collection section (page 11 - 15) detailing data points]**
	18b	Plans to promote participant retention and complete follow-up, including list of any outcome data to be collected for participants who discontinue or deviate from intervention protocols<br>**[Withdrawal from study (page 17)]**
Data management	19	Plans for data entry, coding, security, and storage, including any related processes to promote data quality (eg, double data entry; range checks for data values). Reference to where details of data management procedures can be found, if not in the protocol<br>**[Data Sources and Measurements (pages 15 - 16)]**
Statistical methods	20a	Statistical methods for analysing primary and secondary outcomes. Reference to where other details of the statistical analysis plan can be found, if not in the protocol<br>**[Analysis Plan (page 16) and Statistical Methods (page 16 – 17)]**
	20b	Methods for any additional analyses (eg, subgroup and adjusted analyses)<br>**[Statistical Methods (page 16 – 17)]**
	20c	Definition of analysis population relating to protocol non-adherence (eg, as randomised analysis), and any statistical methods to handle missing data (eg, multiple imputation)<br>**[Statistical Methods (page 16 – 17)]**
**Methods: Monitoring**		
Data monitoring	21a	Composition of data monitoring committee (DMC); summary of its role and reporting structure; statement of whether it is independent from the sponsor and competing interests; and reference to where further details about its charter can be found, if not in the protocol. Alternatively, an explanation of why a DMC is not needed<br>**[Quality Control Measures (page 17)]**

	21b	Description of any interim analyses and stopping guidelines, including who will have access to these interim results and make the final decision to terminate the trial **[N/A]**
Harms	22	Plans for collecting, assessing, reporting, and managing solicited and spontaneously reported adverse events and other unintended effects of trial interventions or trial conduct **[Adverse Events Reporting Statement (page 18)]**
Auditing	23	Frequency and procedures for auditing trial conduct, if any, and whether the process will be independent from investigators and the sponsor **[On-going audit from Sponsor's Research Department which is independent from the research team]**
**Ethics and dissemination**		
Research ethics approval	24	Plans for seeking research ethics committee/institutional review board (REC/IRB) approval **[Ethics Committee Approval Statement (page 19)]**
Protocol amendments	25	Plans for communicating important protocol modifications (eg, changes to eligibility criteria, outcomes, analyses) to relevant parties (eg, investigators, REC/IRBs, trial participants, trial registries, journals, regulators) **[Any study ethics amendments will be communicated internally with all the investigators and collaborators. Study registration will be updated]**
Consent or assent	26a	Who will obtain informed consent or assent from potential trial participants or authorised surrogates, and how (see Item 32) **[Recruitment (pages 10 – 11)]**
	26b	Additional consent provisions for collection and use of participant data and biological specimens in ancillary studies, if applicable **[N/A]**
Confidentiality	27	How personal information about potential and enrolled participants will be collected, shared, and maintained in order to protect confidentiality before, during, and after the trial **[Data Sources and Measurements (page 15 – 16)]**
Declaration of interests	28	Financial and other competing interests for principal investigators for the overall trial and each study site **[Funding Statement and Competing Interests Statement (page 21)]**

For peer review only

Access to data	29	Statement of who will have access to the final trial dataset, and disclosure of contractual agreements that limit such access for investigators<br>**[Author Contributions (page 20) and Competing Interests (page 21)]**
Ancillary and post-trial care	30	Provisions, if any, for ancillary and post-trial care, and for compensation to those who suffer harm from trial participation<br>**[N/A]**
Dissemination policy	31a	Plans for investigators and sponsor to communicate trial results to participants, healthcare professionals, the public, and other relevant groups (eg, via publication, reporting in results databases, or other data sharing arrangements), including any publication restrictions<br>**[Dissemination Policy (Page 18)]**
	31b	Authorship eligibility guidelines and any intended use of professional writers<br>**[N/A]**
	31c	Plans, if any, for granting public access to the full protocol, participant-level dataset, and statistical code<br>**[Full protocol available online at ClinicalTrials.gov (details on page 21)]**
**Appendices**		
Informed consent materials	32	Model consent form and other related documentation given to participants and authorised surrogates<br>**[N/A]**
Biological specimens	33	Plans for collection, laboratory evaluation, and storage of biological specimens for genetic or molecular analysis in the current trial and for future use in ancillary studies, if applicable<br>**[N/A]**

*It is strongly recommended that this checklist be read in conjunction with the SPIRIT 2013 Explanation & Elaboration for important clarification on the items. Amendments to the protocol should be tracked and dated. The SPIRIT checklist is copyrighted by the SPIRIT Group under the Creative Commons "Attribution-NonCommercial-NoDerivs 3.0 Unported" license.

For peer review only
